# Clinical Insights Into Aortitis Following Granulocyte Colony-Stimulating Factor Use in an Older Male Patient

**DOI:** 10.7759/cureus.85801

**Published:** 2025-06-11

**Authors:** Kaichi Sugihara, Takahiro Kamihara, Takuya Omura, Atsuya Shimizu

**Affiliations:** 1 Department of Urology, Japanese Red Cross Aichi Medical Center, Nagoya Daini Hospital, Nagoya, JPN; 2 Department of Cardiology, National Center for Geriatrics and Gerontology, Obu, JPN; 3 Department of Endocrinology and Metabolism, National Center for Geriatrics and Gerontology, Obu, JPN

**Keywords:** aortitis, fever, fever of unknown, granulocyte colony-stimulating factor (g-csf), older adult

## Abstract

This report describes a rare case of extensive aortitis associated with granulocyte colony-stimulating factor (G-CSF) administration in a 74-year-old male with castration-resistant prostate cancer and multiple metastases. The patient developed recurrent fever and elevated inflammatory markers after receiving filgrastim for docetaxel-induced neutropenia. Despite antibiotic treatment, his fever persisted. A contrast-enhanced CT scan revealed circumferential wall thickening of the common carotid artery to the aortic arch and abdominal aorta, prompting suspicion of G-CSF-associated vasculitis. Following the initiation of prednisolone, the patient's fever resolved, inflammatory markers decreased, and clinical status improved. Subsequent CT scans showed a reduction in aortic wall thickening. This case highlighted that G-CSF-associated vasculitis can occur in older male cancer patients, urging clinicians to consider aortitis in the differential diagnosis for unexplained fever and inflammation following G-CSF administration and promptly perform contrast-enhanced CT when suspected.

## Introduction

This report details a rare case of extensive aortitis associated with granulocyte colony-stimulating factor (G-CSF) administration in an older male patient. Given the life-threatening nature of aortitis and its risk of aortic dissection, early diagnosis is paramount [1,2]. To underscore the critical importance of prompt diagnosis and to disseminate knowledge about its diagnostic approaches across various medical specialties, we present this case report. This report is particularly significant due to the rarity of reported cases of G-CSF-induced aortitis in older male cancer patients.

## Case presentation

The patient, a 74-year-old male with a history of localized prostate cancer treated with bicalutamide and androgen deprivation therapy 10 years prior, presented with a gradual elevation in prostate-specific antigen (PSA) levels (0.066 ng/mL) and subsequent pulmonary metastases. A bronchoscopic lung biopsy confirmed adenocarcinoma, with PSA levels further escalating to 0.156 ng/mL. The treatment regimen was altered to enzalutamide; however, PSA levels remained elevated, and progressive pulmonary metastases and new bone metastases were observed. Consequently, the elevated PSA level indicated systemic metastasis, leading to the patient's admission for docetaxel induction therapy for castration-resistant prostate cancer with pulmonary and bone metastases. His medical history was notable for diabetes mellitus, hyperlipidemia, and stable angina pectoris (post-catheterization), all managed pharmacologically. Admission blood tests revealed no significant abnormalities beyond elevated PSA levels (Table 1).

**Table 1:** Laboratory findings on admission.

Hematology			
Parameter	Patient Value	Unit	Reference Range
White Blood Cell Counts	4700	/μL	3300-8600
Neutrophils	49.2	%	38-68
Lymphocytes	39.8	%	27-47
Monocytes	8.7	%	2.0-8.0
Eosinophils	2.1	%	2.0-8.0
Basophils	0.2	%	0.0-7.0
Red Blood Cell Counts	4.87×10^6^	/μL	4.35-5.55×10^6^
Hemoglobin	12.1	g/dL	13.7-16.8
Hematocrit	39	%	40.7-50.1
Mean Corpuscular Volume	80.1	fL	83.6-98.2
Mean Corpuscular Hemoglobin	24.8	pg	27.5-33.2
Platelet	24.7×10^3^	/μL	15.8-34.8×10^3^
Biochemistry			
Total Protein	7.2	g/dL	6.6-8.1
Albumin	4	g/dL	4.1-5.1
Total Bilirubin	0.2	mg/dL	0.4v1.5
Aspartate Aminotransferase	15	IU/L	13-30
Alanine Transaminase	11	IU/L	10-42
Alkaline Phosphatase	133	U/L	38-113
Lactate Dehydrogenase	182	U/L	124-222
Glucose	114	mg/dL	44-132
Uric Acid	4	mg/dL	73-109
Urea Nitrogen	21	mg/dL	73-109
Creatinine	0.8	mg/dL	0.65-1.07
Estimated Glomerular Filtration Rate	72	mL/min/1.73 m^2^	90-
Sodium	142	mEq/L	138-145
Potassium	4.4	mEq/L	3.6-4.8
Chlorine	105	mEq/L	101-108
Calcium	10.1	mg/dL	8.8-10.1
Serology			
C-reactive Protein	0.42	mg/dL	0.00-0.14
Prostate-Specific Antigen	0.464	ng/mL	0.0-4.0

**Figure 1:**
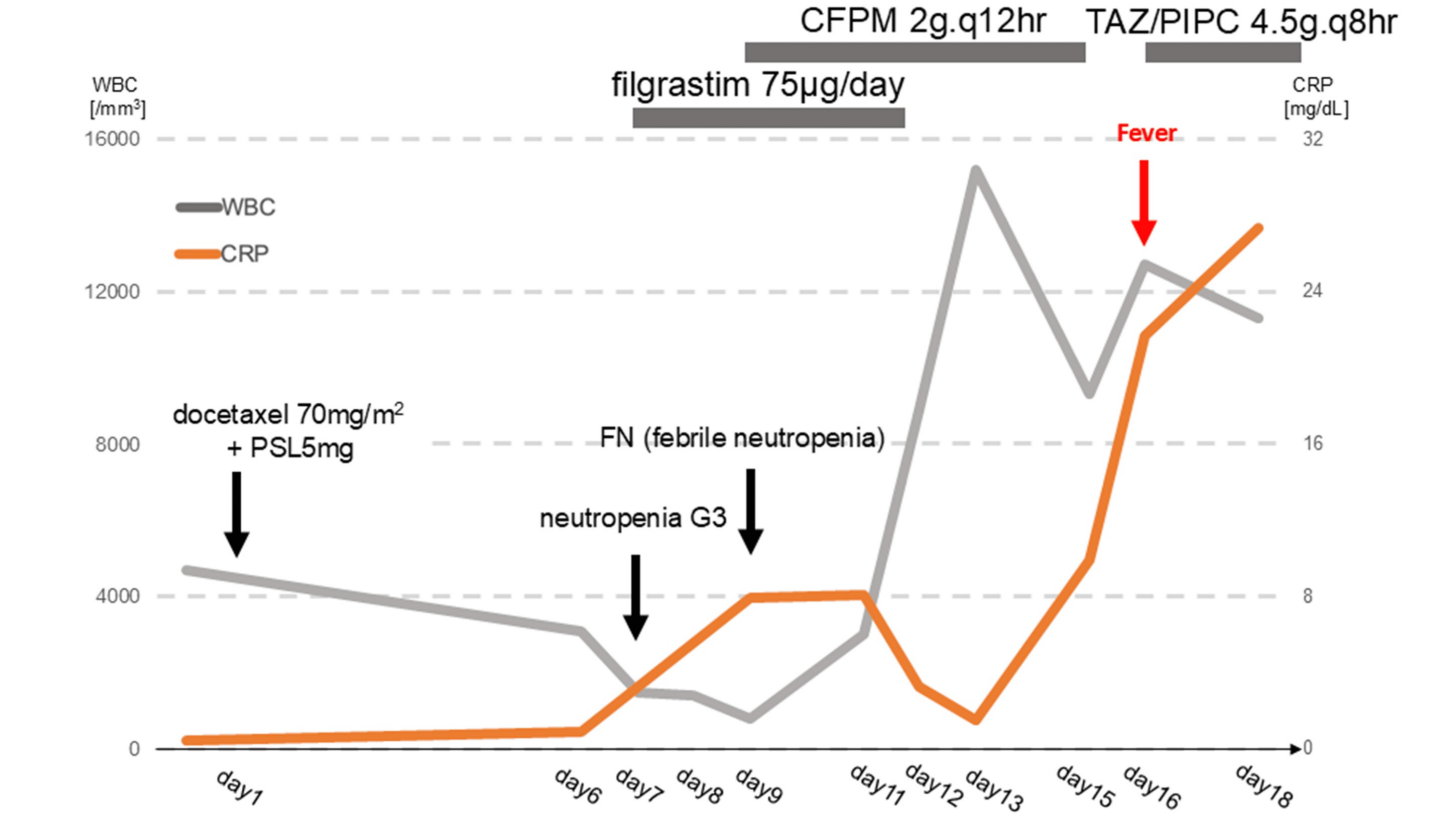
Clinical course leading to the diagnosis of aortitis. Docetaxel (70 mg/m²) and PSL (5 mg) were initiated. On day 7 post-treatment initiation, grade 3 neutropenia was observed, prompting the administration of filgrastim (75 μg). The patient developed FN on day 9, which resolved with cefepime (2 g every 12 hours). Filgrastim was discontinued on day 12 following neutrophil recovery. However, the patient experienced recurrent fever on day 16, accompanied by elevated inflammatory markers (WBC 10,700/μL, CRP 28.2 mg/dL). WBC, white blood cell; CRP, C-reactive protein; CFPM, cefepime; TAZ/PIPC, tazobactam/piperacillin; PSL, prednisolone; FN, febrile neutropenia

Docetaxel (70 mg/m²) and prednisolone (PSL, 5 mg) were initiated. Grade 3 neutropenia was observed on day 7 post-treatment initiation, prompting the administration of filgrastim (75 μg). The patient developed febrile neutropenia (FN) on day 9, which resolved with cefepime (2 g every 12 hours). Filgrastim was discontinued on day 12 following neutrophil recovery. However, the patient experienced recurrent fever on day 16 (Figure 1), accompanied by elevated inflammatory markers (WBC 10,700/μL, CRP 28.2 mg/dL).

CT scans revealed no apparent infectious source, and tazobactam/piperacillin was initiated following blood culture collection. Despite this, the patient remained febrile, and inflammatory markers persisted at elevated levels (Table 2). This blood sample was collected on day 16 after the initiation of chemotherapy.

**Table 2:** Laboratory findings in vasculitis-associated fever. This blood sample was collected on day 16 after the initiation of chemotherapy. WBC, white blood cell; RBC, red blood cell; Hb, hemoglobin; HCT, hematocrit; MCV, mean corpuscular volume; MCH, mean corpuscular hemoglobin; TP, total protein; Alb, albumin; T-BIL, total bilirubin; AST, aspartate aminotransferase; ALT, alanine transaminase; ALP, alkaline phosphatase; LDH, lactate dehydrogenase; Glu, glucose; UA, uric acid; UN, urea nitrogen; Cre, creatinine; eGFR, estimated glomerular filtration rate; Na, sodium; K, potassium; Cl, chlorine; Ca, calcium; Fe, free iron; UIBC, unsaturated iron-binding capacity; TIBC, total iron-binding capacity; PT, prothrombin time; PT-INR, prothrombin time-international normalized ratio; APTT, activated partial thromboplastin time; FDP, fibrinogen/fibrin degradation product; CRP, C-reactive protein; PCT, procalcitonin; ANA, antinuclear antibody; MPO-ANCA, myeloperoxidase-anti-neutrophil cytoplasmic antibody; PR3-ANCA, proteinase 3-anti-neutrophil cytoplasmic antibody; CCP, cyclic citrullinated peptide

Hematology			
Parameter	Patient Value	Unit	Reference Range
WBC	11300	/μL	3300-8600
Neutrophils	84.5	%	38-68
Lympocytes	9.1	%	27-47
Monocytes	6.3	%	2.0-8.0
Eosinophils	0	%	2.0-8.0
Basophils	0.1	%	0.0-7.0
RBC	3.94×10^6^	/μL	4.35-5.55×10^6^
Hb	9.6	g/dL	13.7-16.8
HCT	31.2	%	40.7-50.1
MCV	79.2	fL	83.6-98.2
MCH	24.4	pg	27.5-33.2
Platelet	23.9×10^3^	/μL	15.8-34.8×10^3^
Biochemistry			
TP	6.5	g/dL	6.6-8.1
Alb	2.8	g/dL	4.1-5.1
T-BIL	0.4	mg/dL	0.4-1.5
AST	19	IU/L	13-30
ALT	19	IU/L	10-42
ALP	114	U/L	38-113
LDH	159	U/L	124-222
GLU	79	mg/dL	44-132
UA	2.4	mg/dL	73-109
UN	15	mg/dL	73-109
Cre	0.82	mg/dL	0.65-1.07
eGFR	70.1	mL/min/1.73m^2^	90-
Na	138	mEq/L	138-145
K	4.2	mEq/L	3.6-4.8
Cl	103	mEq/L	101-108
Ca	8.3	mg/dL	8.8-10.1
Fe	15	μg/dL	40-188
UIBC	175	μg/dL	168-252
TIBC	190	μg/dL	222-433
Ferritin	627.4	ng/mL	21.8-274.7
Coagulation			
PT	15.1	sec	9.8-12.5
PT-INR	1.35		0.9-1.1
APTT	34.4	sec	30.0-40.0
FDP	1.1	μg/mL	0.0-5.0
D-dimer	4.7	μg/mL	0.0-1.0
Serology			
CRP	27.33	mg/dL	0.00-0.14
PCT	0.27	ng/mL	0.00-0.05
C3	163	mg/dL	73-138
C4	37	mg/dL	11-31
IgG	1007	mg/dL	861-1747
IgG4	26	mg/dL	11-121
IgA	554	mg/dL	93-393
IgM	46	mg/dL	33-183
ANA	<40		0-39
Anti-Jo-1 Antibody	<0.3	U/mL	0.0-6.9
Anti-RNP Antibody	3.1	U/mL	0.0-3.5
Anti-Sm Antibody	<0.7	U/mL	0.0-7.0
Anti-Scl-70 Antibody	0.7	U/mL	0.0-7.0
Anti-SS-A/La Antibody	1.7	U/mL	0.0-7.0
Anti-SS-B/La Antibody	<0.4	U/mL	0.0-6.9
Anti-dsDNA Antibody	2	IU/mL	0-10
Anti-ssDNA Antibody	3	U/mL	0-7
Anti-Cardiolipin Antibody	<4.0	U/mL	0.0-12.3
MPO-ANCA	<0.2	IU/mL	0.0-3.4
PR3-ANCA	<0.5	IU/mL	0.0-2.0
Anti-CCP Antibody	<0.5	U/mL	0.0-4.4
Urinalysis			
pH	6		5.5-7.5
Protein	( - )		( - )
Glucose	( - )		( - )
RBC	20-29	/HPF	( - )
WBC	10-19	/HPF	( - )

**Figure 2:**
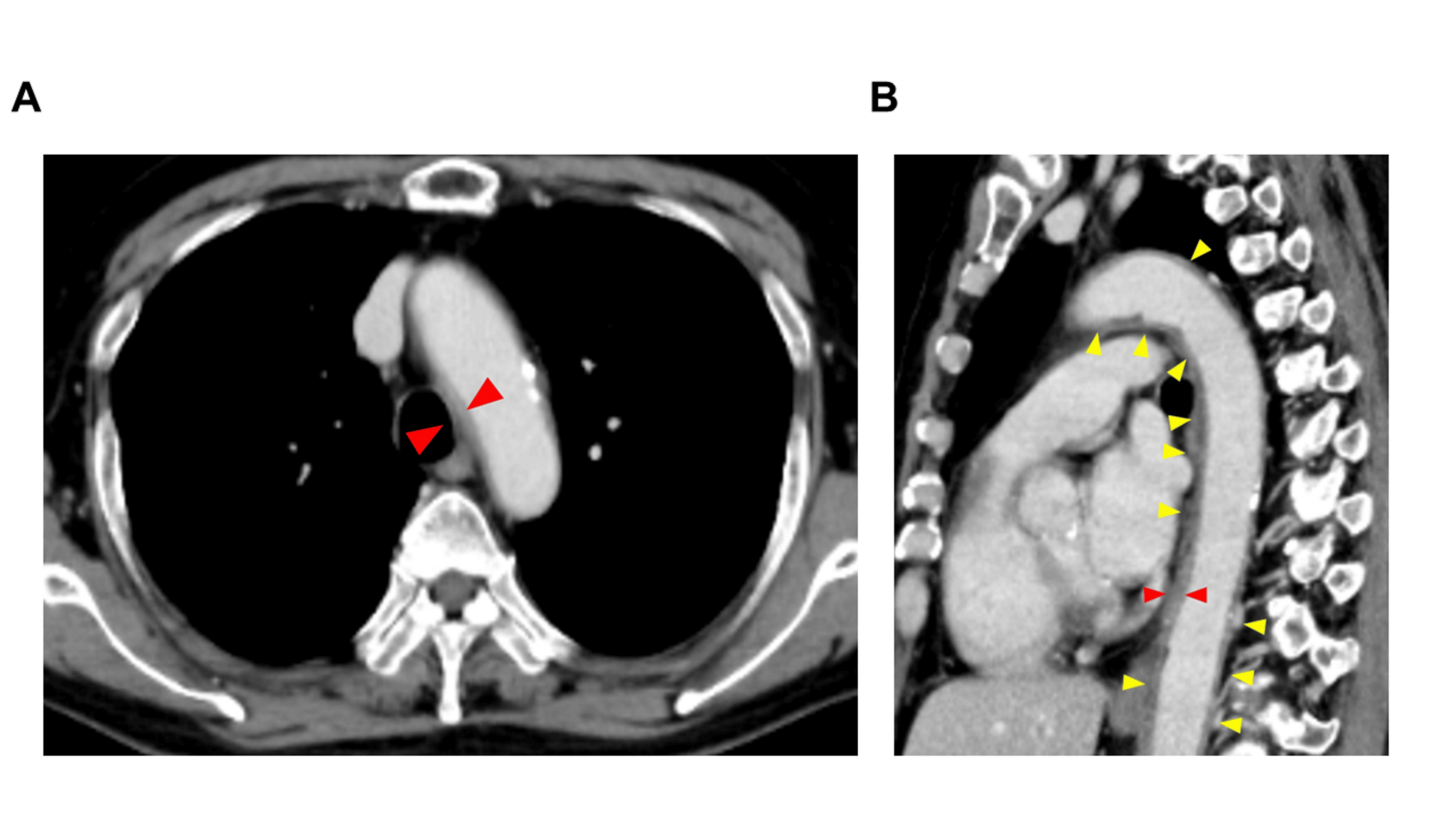
(A) Contrast-enhanced CT at diagnosis: a horizontal view. (B) Contrast-enhanced CT at diagnosis: a sagittal view. (A) This figure shows a horizontal view from a contrast-enhanced CT scan performed on day 19 after docetaxel initiation to identify the fever source, revealing extensive circumferential wall thickening from the common carotid artery to the aortic arch and abdominal aorta. (B) This figure displays a sagittal view. Yellow arrows indicate the extent of aortic inflammation at the time of aortitis diagnosis. Red arrows are used to compare the aortic wall thickness.

A contrast-enhanced CT on day 19, performed for fever source localization, revealed circumferential wall thickening of the common carotid artery to the aortic arch and abdominal aorta (Figure 2).

**Figure 3:**
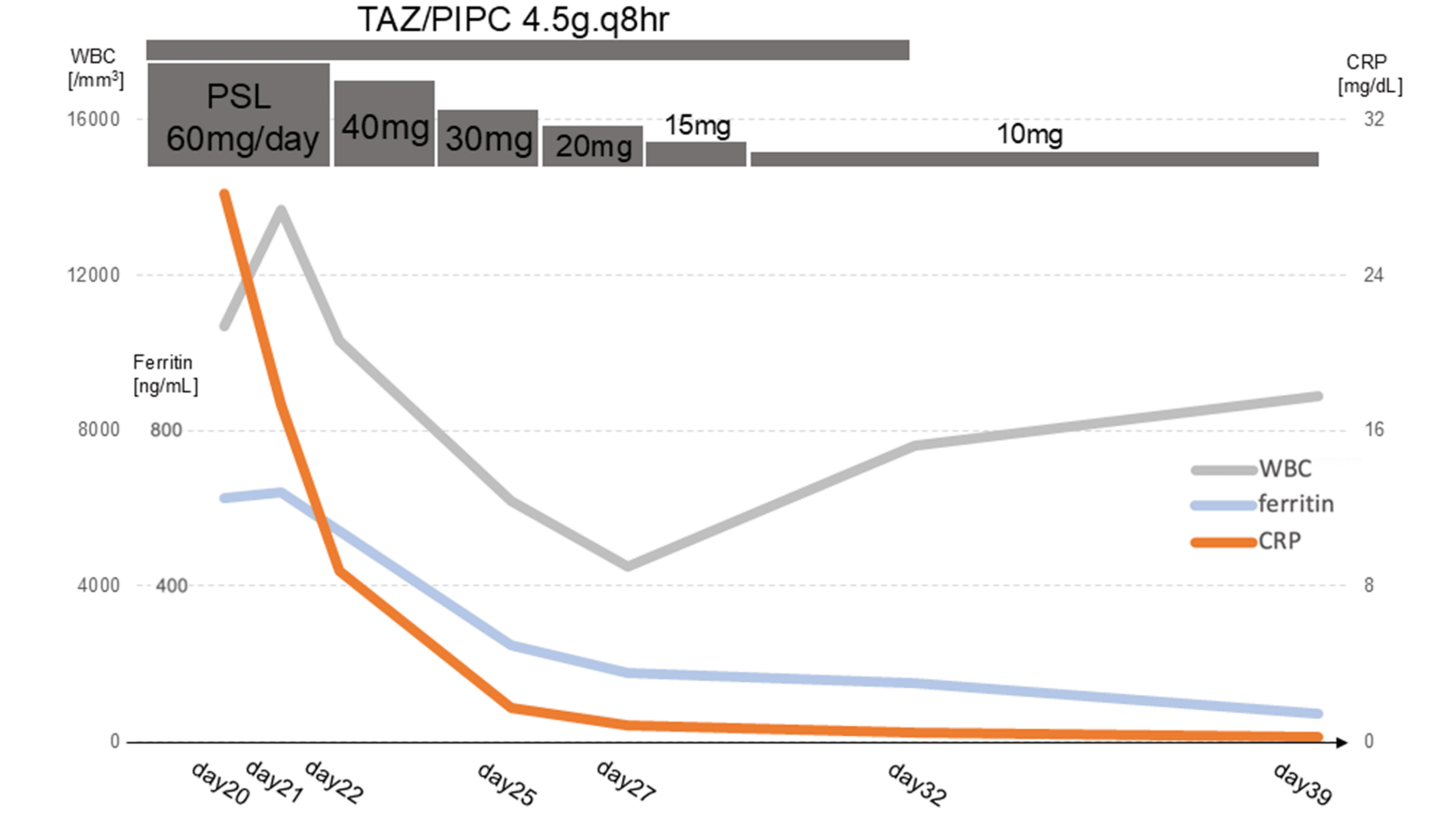
Clinical course following initiation of steroid therapy. After the commencement of steroid treatment, the patient's fever resolved, inflammatory markers decreased, and overall clinical condition improved. PSL was tapered to 10 mg over two weeks, and the patient was discharged on day 32 without a recurrence of fever or elevated inflammatory markers. WBC, white blood cell; CRP, C-reactive protein; TAZ/PIPC, tazobactam/piperacillin; PSL, prednisolone

Given the absence of bacterial growth in blood cultures and the patient's refractoriness to antibiotics, an infectious etiology was deemed improbable. Furthermore, the low epidemiological likelihood of Takayasu arteritis or giant cell arteritis led to the suspicion of drug-induced vasculitis, specifically G-CSF-associated vasculitis. PSL (60 mg, 1 mg/kg/day) was initiated. Moreover, IgG4-related periaortitis was deemed unlikely due to the lack of elevated IgG4. The patient's autoantibody profile was negative (Table 2), consistent with drug-induced vasculitis. Although some test results were near the upper limit of normal, the symptoms were nonspecific and not considered diagnostic. Following steroid initiation, the patient's fever resolved, inflammatory markers decreased, and overall clinical status improved (Figure 3).

**Figure 4:**
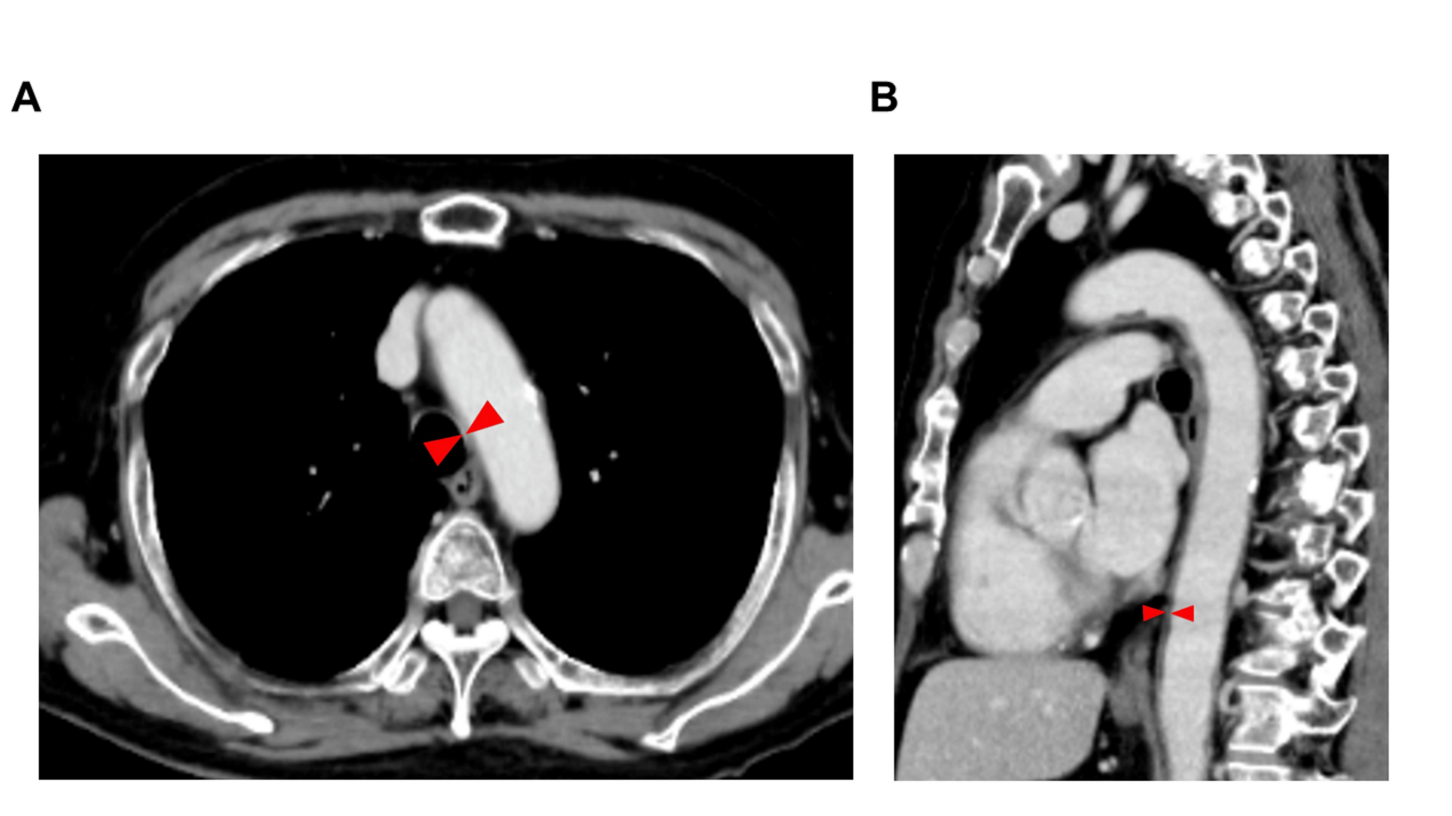
(A) Progression of findings on contrast-enhanced CT: a horizontal view. (B) Progression of findings on contrast-enhanced CT: a sagittal view. (A) A subsequent CT scan post-third docetaxel cycle (day 39 from initial docetaxel) demonstrated sustained reduction of aortic wall thickening. (B) This figure displays a sagittal view.  Red arrows are used to compare the aortic wall thickness.

As continued high-dose steroid therapy was undesirable, we aimed for steroid dose reduction. PSL was tapered to 10 mg over two weeks, and the patient was discharged on day 32 without a recurrence of fever or elevated inflammatory markers. A follow-up contrast-enhanced CT on day 39 showed a reduction in aortic wall thickening (Figure 4).

The second docetaxel cycle was initiated with PSL maintained at 10 mg and docetaxel reduced to 55 mg/m². A subsequent CT scan post-third docetaxel cycle demonstrated a sustained reduction of aortic wall thickening. In this case, three chemotherapy cycles were completed through increased steroid dosage (5 mg to 10 mg), reduced docetaxel dosage, and prophylactic antibiotic administration for FN. Aortitis has not been confirmed for seven months following that event. Unfortunately, docetaxel was later discontinued due to a lack of therapeutic efficacy in treating the cancer. However, blood tests and other monitoring continue to be performed regularly to rule out any recurrence of aortitis.

## Discussion

This case report describes a patient who developed G-CSF-associated aortitis during chemotherapy but subsequently completed three additional cycles without a recurrence of vasculitis. While we initially considered a risk of G-CSF-associated aortitis recurrence if docetaxel had been continued without dose reduction, the successful completion of chemotherapy without vasculitis relapse was ultimately achieved by co-administering PSL with docetaxel and reducing the docetaxel dose to 55 mg/m² in the second cycle.

Although cabazitaxel would typically be the next treatment course after docetaxel, we determined that administering cabazitaxel without concurrent G-CSF would be risky due to its higher risk of FN compared to docetaxel. Discussions regarding alternative options to filgrastim for recurrent neutropenia are also necessary. In such cases, prophylactic levofloxacin may be recommended for persistent neutropenia below 100/µL. We believe this case report will be valuable for many clinicians involved in the future management of patients who develop aortitis during chemotherapy, especially internists, oncologists, and other physicians in cancer-related specialties, general internists managing fevers of unknown origin, and geriatricians caring for various conditions in older adults.

Aortitis should be included in the differential diagnosis in patients presenting with fever and inflammation following G-CSF administration. While G-CSF-associated vasculitis is more commonly reported in females, breast cancer cases, and those with other gynecological conditions [3], this case highlights the occurrence of G-CSF-associated vasculitis in older males. A review of the extant literature indicates that the incidence of G-CSF-associated vasculitis ranges from 0.4% to 2.7% [4-6]. However, subsequent reports, including this case, on prostate and bladder cancer patients not featured in those original studies indicate that this vasculitis may manifest across a broader spectrum of age, gender, and cancer types than previously recognized. Cases of G-CSF-associated aortitis in older patients, specifically those with bladder and prostate cancer, have only recently started to be reported [7,8]. In cancer patients, particularly older individuals, fever evaluation can be challenging due to nonspecific symptoms. Furthermore, secondary aortitis may be asymptomatic despite progressive vascular inflammation. This case underscores the importance of considering G-CSF-associated aortitis in older males and the necessity for prompt contrast-enhanced CT imaging when G-CSF-associated vasculitis is suspected. The clinical lesson from this case is that aortitis should be considered a complication in any cancer treatment, and if suspected, contrast-enhanced CT should be performed immediately.

## Conclusions

This case report clearly delineated the disease characteristics and treatment approaches that were crucial for healthcare professionals managing cancer and those investigating fevers of unknown origin. This case report, by detailing the clinical course of an older male patient and referencing prior literature, contributes to the understanding that aortitis, previously considered more prevalent in gynecological oncology patients receiving chemotherapy, must now be considered in the differential diagnosis for malignancies occurring in older men.
